# Energy Efficiency in CO_2_ Laser Processing of Hardox 400 Material

**DOI:** 10.3390/ma15134505

**Published:** 2022-06-26

**Authors:** Constantin Cristinel Girdu, Catalin Gheorghe

**Affiliations:** 1 Department of Manufacturing Engineering, Transilvania University of Brasov, Eroilor Street 29, 500036 Brasov, Romania; girdu.constantin.cristinel@unitbv.ro; 2 Department of Engineering and Industrial Management, Transilvania University of Brasov, Eroilor Street 29, 500036 Brasov, Romania

**Keywords:** cutting efficiency, linear energy, HARDOX 400, CO_2_ laser, ANOVA, RSM

## Abstract

The use of laser technology for materials processing has a wide applicability in various industrial fields, due to its proven advantages, such as processing time, economic efficiency and reduced impact on the natural environment. The expansion of laser technology has been possible due to the dynamics of research in the field. One of the directions of research is to establish the appropriate cutting parameters. The evolution of research in this direction can be deepened by determining the efficiency of laser cutting. Starting from such a hypothesis, the study contains an analysis of laser cutting parameters (speed, power and pressure) to determine the linear energy and cutting efficiency. For this purpose, the linear energy and the cutting efficiency were determined analytically, and the results obtained were tested with the Lagrange interpolation method, the statistical mathematical method and the graphical method. The material chosen was Hardox 400 steel with a thickness of 8 mm, due to its numerous industrial applications and the fact that it is an insufficiently studied material. Statistical data processing shows that the maximum cutting efficiency is mainly influenced by speed, followed by laser power. The results obtained reduce energy costs in manufacturing processes that use the CO_2_ laser. The combinations identified between laser speed and power lead to a reduction in energy consumption and thus to an increase in processing efficiency. Through the calculation relationships established for linear energy and cutting efficiency, the study contributes to the extension of the theoretical and practical basis.

## 1. Introduction

The concern regarding cost reduction drives all companies regardless of their business object. Increasing interest in development costs in the past few years is guided by the consumption of raw materials, materials and energy and rising prices for their use, processing and transportation, investment in research, limited reserves of production factors and the depletion of natural resources. Another key element for the costs of economic development is the environment which, in order to support development, must be in the right balance. In view of these challenges, the manufacturing industry has oriented towards more economical processing, characterized by lower energy consumption, raw materials and environmentally friendly materials, and the elimination of the resulting waste.

One of the modern processes that is part of these trends is laser processing. The use of this technology has made it possible to obtain processes such as cutting, engraving, drilling, welding, directed energy deposition, alloying, brazing and scribing [1,2]. Due to its economic, technological, and environmental advantages, laser processing has developed rapidly. It is currently applied in several industries such as automotive, aeronautics, pharmaceuticals and extractive [3,4]. It appears as a sustainable alternative, an environmentally friendly processing process, capable of generating high quality surfaces [4,5]. In addition, rising commodity and energy prices in the past few months and future prospects are accelerating such a process [5,6].

Hardox 400 steel, on which the cutting experiments were performed, has high wear and corrosion resistance, a good ratio between strength and weight and proper cold plastic deformation properties. Due to its special mechanical and thermal properties, steel is used in various industries, for applications that combine abrasion resistance with good cold bending properties [7,8]. Hardox 400 steel has better corrosion resistance than other steels, is resistant to fatigue, has good resistance to impact, good weldability and it is not intended for an additional thermal treatment [7,9].

The use of classical technologies for machining Hardox 400 steel results in rapid tool wear and average roughness of the surfaces obtained [10,11]. Hardox 400 steel processing is difficult due to the thermal conductivity, reflectivity and viscosity of the molten material [12,13]. As a result of these difficulties, laser cutting becomes an alternative process for efficient processing of Hardox 400 steel [4,13,14,15].

Laser beam machining allows us to obtain parts with a complex geometric shape. In the field of industrial manufacturing such a process consists in concentrating the laser radiation on the surface of the metal target. The energy of the incident radiation is distributed with the help of a laser spot with a 0.2 mm diameter. By irradiation, the spot heats up and melts almost instantly, a very small area of material with a penetration depth, equal to the diameter of the focal spot. The result is a piece of material called melt which, by moving through the material, produces the operation of cutting the part. Laser cutting of metals is conducted with the laser beam surrounded by the assistant gas that evacuates the melt. There is a convective heat exchange between the layers of molten material. A heat diffusion through the material takes place through thermal conduction. Energy is consumed for heating to the melting temperature, which can be analytically estimated according to the physical and thermal characteristics of the material. Using a continuous laser pulse by irradiation, a penetration of the material takes place, and by moving the laser spot at a constant speed, the cutting is performed. The state parameters that characterize the technological cutting process are calculated according to the laser spot characteristics, the thermal and physical properties of the material. When laser radiation interacts with metals, the laser energy develops additional heat, produced by the oxidation reaction that burns the material and determines the cutting width.

The paper aims to determine and model the efficiency of laser radiation processing. An important measure in the field of laser processing is energy. This plays an important role in cutting because, starting from this concept, other sizes can be defined to describe the technological process of cutting. An example is the cutting efficiency (E_c_), meaning the side surface of the workpiece machined with 1 Joule:

(1) $$E_{c}=\frac{A}{E}[\frac{{\mathrm{mm}}^{2}}{J}],$$

where A is the side area of the piece, and E is the laser energy transformed into the heat needed to melt the material. Another measure that describes the cut is the melting efficiency (E_m_). The concept is useful to determine the efficiency of the cutting process. The indicator can be set as the volume of the molten material with an energy consumption equal to 1 Joule.

(2) $$E_{m}=\frac{V}{E}[\frac{{\mathrm{mm}}^{3}}{J}],$$

where V is the molten volume. Melting efficiency can be defined as the product of cutting efficiency and Kerf.

Another measure used in research, defined by the authors, is linear energy (E_l_). The mathematical relationship is given by the energy of the laser relative to the unit of length of the piece (l).

(3) $$E_{l}=\frac{E}{l}\;[\frac{J}{\mathrm{mm}}].$$

The purpose of the manuscript is to determine the efficiency of the laser radiation processing. Laser heat processing technology has as its objective factor the spot, in some works the focus. The laser spot consists of a focal spot with a radius of up to a few tens of microns where the laser intensity is maximum. The transmission of laser radiation to the surface of the material takes place through a converging lens. The laser spot interferes with all the refracted rays passing through the lens. This light source emits a very small laser beam of divergence. In the place where the laser radiation meets the metal, a liquid area is produced, which is removed from the assistant gas jet. The processing conditions are due to the intensity of the laser spot, the interaction time between radiation and metal and the thermal and physical properties of the material.

## 2. Synthesis of Literature

In the past few years, researchers studied the possibility of cutting with a CO_2_ laser of different materials such as methyl polymethacrylate [16], Al 6061–T6 alloy [17], 304 stainless steel [18], AISI 1045 austenitic steel [19], AlSI 304, AlMg3 and St37-2 [20], SS41 and SUS304 [21], pure titanium and Ti-6Al-4V titanium alloys [22], Ti6Al4V alloy [23] and Nickel-based super alloys Nimonic 263 [24].

Naik and Maity (2020) conducted an experimental investigation of cutting 10 mm thick Hardox 400 boards using various gases (air, argon, oxygen and nitrogen). The experiments were performed on a numerically controlled plasma cutting machine. The results refer to the influence of each gas used on the workpiece [3]. Ramos et al. followed the surface condition influence before processing on the cut parts quality. For this purpose, the authors used Hardox steel plates, pickled steel and Ruukki steel with thicknesses of 6 mm, 10 mm and 15 mm, which they cut with a CO_2_ laser. The results highlight differences in quality (top, centre and bottom) between pickled steel and Hardox [12]. The analysis of the literature confirms that the number of works that addressed Hardox 400 steel is low.

Most of research has focused on establishing a set of parameters that lead to the optimization of cutting width, surface quality, and speed of cutting. Anghel et al. analysed the CO_2_ cutting of 304 stainless steel with laser beam. The authors aimed to find the roughness of the cut surfaces R_a_ as small as possible. The parameters studied using the RSM (response surface model) were focal position, cutting speed, power and auxiliary gas pressure. Using statistical tools, the authors demonstrate that, among the studied parameters, the focal position has a decisive influence [18]. Genna et al. provided a comparative analysis of three materials, AlMg3, St37-2 and AlSI 304, in terms of laser cutting width. The auxiliary gases used in the experimental design were N_2_ and O_2_. The authors analysed the combinations of cutting parameters that have the effect of minimizing the cutting width. The results show that kerf decreases if the cutting speed is higher, and the heat accumulation is lower [20]. Levichev et al. performed laser cutting experiments with a power set at 4 kW, on a 15 mm thick steel plate, the auxiliary gas being oxygen. The aim was to reduce the material losses, generated by the continuous heating of the steel plate during cutting. The authors showed that, in areas where the cutting speed is low, material loss occurs and the quality of the cut decreases. To eliminate these shortcomings, the authors propose the optimization of the cutting parameters and cooling of the semi-finished product, so that the excess heat is eliminated [25]. We find the same orientation of research in Madic et al. The authors proposed an optimal combination of CO_2_ laser cutting parameters that leads to quality cut surfaces and lower costs. The experimental design was made on material from the steel industry and pursued a maximum melt removal speed [26]. Jiang et al. considered, in the research conducted, several process parameters that characterize laser cutting. These parameters were cutting speed, laser power, the distance between nozzle, and blank, sheet thickness, and temperature in the cutting area. The experimental material was Al 6061–T6 alloy. The results obtained by the authors show that the values of the process parameters are determined by the thickness of the alloy sheet. According to the authors, power is the parameter with a high influence on the quality of the cut surface. In addition, the change in power density has the effect of varying the temperature in the cutting area [17].

Pramanik et al. considered as working parameters the laser power, the Z distance, the cutting angle, the pulsation frequency and the scanning speed. The authors studied their influence on cutting width, and R_a_ surface roughness. The material used was 1 mm thick titanium plates [27]. Das Partha et al. considered the research carried out the cutting speed, the gas pressure, the pulsation frequency, and the pulse width. Using two solid Nd, YAG laser sources and pulsed CO_2_ gas, they identified the optimal combination that leads to a low R_a_ roughness, and cutting width [28]. Nath et al. followed the influence of speed and power laser on depth, thermal stress, and cutting width. The authors have shown that the cutting depth varies in direct proportion to the laser power, and the thermal stress varies depending on the cutting parameters. The speed increase reduces the kerf, which leads to material saving in the case of functionally graded materials (FGMs) [29]. Chen et al. considered in the research on AISI 1045 steel, the following input parameters laser power, focusing distance and scanning speed. Statistical processing of the data led to the conclusion that, after laser cutting the hardness of the hardened surface increases from 200 HV to 660 HV [19]. El Aoud et al. recommended CO_2_ laser cutting for pure titanium and Ti-6Al-4V titanium alloy sheets. For both materials, the authors demonstrated statistically, that laser power is the most significant cutting parameter, and cutting width is inversely proportional to the speed [22]. A similar conclusion was reached by Chatterjee et al. when studying laser drilling of Ti6Al4V alloy sheets. In order to generate holes with quality surface and good taper, the laser power must be set correctly, as it is the parameter with the greatest influence [23]. Kang et al. used ultrafast lasers in the production of WC-Co alloy steel cutting tools. The experiments were performed by modifying the assistant gas pressure, wavelength and fluency. The authors found that the laser with different wavelengths 532 nm (VIS) and 1064 nm (IR) can be used to round the cut edges, because the accuracy is high. When analysing the mechanical properties of the obtained cutting tools, no change in hardness was observed, and the roughness obtained R_a_ was less than 0.15 µm [30]. Sibalija et al. analysed the following parameters: cutting speed, auxiliary gas pressure, and laser power focusing position on the 2 mm thick Nimonic 263 alloy. The authors optimized the cutting parameters and determined the combinations that lead to an increase in the quality of the cutting area, through use of the method of artificial neural networks [24]. Eltawahni et al. applied a Box Behneken method to CO_2_ laser cutting of AISI316L stainless steel. The parameters studied by the authors were cutting speed, focusing position, nozzle diameter, laser power, and assistant gas pressure in order to statistically model the R_a_ surface roughness, cutting width and machining cost. The results obtained by the authors consist of optimal combinations of input parameters that lead to a high quality of processing and a low cost [31]. Son et al. studied the cutting width for sheet metal SS41 and SUS 304. Through the ANOVA procedure, the authors demonstrated that the significant indicators are the cutting speed and the laser power [21]. Savanth et al. used a YAG laser to apply a layer of Colmonoy-5 to the surface of carbon steel plates. The authors looked at the effect of different combinations of the two parameters (cutting speed and laser power) on micro hardness and wear resistance. The results showed that the laser power has an increased influence on the micro hardness. If the laser power is low and the cutting speed is high, then the wear resistance increases [32]. Moradi et al. considered as input parameters focal position and laser scanning speed, for additive deposition in the manufacture of various parts. The statistical processing of the experimental data obtained, allowed to establish the optimal combinations that lead to the minimization of the kerf [33]. As shown in the previous literature analysis, the input parameters commonly used for sizing the output quantities are cutting speed, laser power and pressure of auxiliary gas. 

Other research has focused on the components involved in laser cutting of various materials. Thus, Seong et al. investigated the behaviour of the gas flow inside the nozzle. The authors used a 6 KW laser power to cut thick sheets of stainless steel [34]. Subasi et al. studied a laser beam arranged through a stream of water to track a micro-drilling process. The materials used were nickel alloys frequently used in the aeronautical domain [35]. 

The analysis of the literature shows that there are few authors who have studied the heat released during laser cutting. Tatzel et al. studied the influence of the focusing position on how heat affects the cut edges of a 3 mm thick steel plate. The authors looked at the influence of the speed, the pressure of the gas, and the focus position on the processing quality. According to the authors, the position of focus is decisive if quality is pursued [36]. Hajad et al. aimed at accumulating a minimum amount of heat during laser cutting. Starting from this objective, the authors established a method of optimizing the cutting path, based on a genetic algorithm with a search in the vicinity. Moreover, research in this direction has focused on cooling the semi-finished product and removing heat more quickly. [37] Zhu et al. focused on laser drilling of composite materials (CFRP). The authors proposed a new laser drilling method that leads to an energy saving of 78.10% [1]. In the experiments conducted, Naik and Maity (2020) measured the cutting performance and energy balance. Although the experiments were performed for plasma cutting, the amount and material rate of removal (MRR) can be considered indicators [3]. Hlavac et al. completed a study of abrasive water jet cutting on Hardox 400 steel. The authors followed the surface roughness and a 3D analysis for five different cutting speeds [38].

From the above analysis, it can be concluded that CO_2_ laser cutting has not been properly researched in the direction of cutting efficiency. Moreover, from the study of the literature conducted by the authors, the optimization of some input parameters according to energy efficiency, was not achieved.

Laser material treatment is an important field of applications of laser technologies in industry that confirms the relevance of this research paper. Currently, there is a trend to replace CO_2_ lasers with fibre lasers. For this reason, the choice of cutting Hardox steel sheets using a CO_2_ laser is debatable, which is why we have proposed to clarify this issue.

The arguments for selecting the CO_2_ laser machine were due to the strong absorbency of the laser radiation by the Hardox material. The main reason for choosing the laser system is the transfer of thermal energy. Several research works have been identified in this direction. Thus, Steen estimated the removal energy required for the material as a measure of the efficiency of the cutting process [39]. Adelman used a fibre laser with a power of 500 W to cut a thin sheet of Al with a thickness 1 mm and a speed of 90 mm/s, obtaining a specific energy of 3.3 J/mm^2^ [40]. Ready used a CO_2_ laser with a power of 1200 W to cut an Al sheet with a thickness 1 mm at a speed of 50 mm/s resulting in a specific energy 24 J/mm^2^ [41]. It was found that in the thin Al board the fibre laser is preferred to the CO_2_ laser due to the fact that it is more efficient by using the low laser power and the increased cutting speed, but also the increased energy consumption of the CO_2_ laser for cutting a 1 mm^2^ of material.

For H400 thickness 8 mm, the best cutting efficiency was obtained at the laser power 4900 W and the cutting speed 1900 mm/min, which corresponds to a specific energy 0.32 J/mm^2^. There is a lower energy consumption of the carbon dioxide laser than that used for the fibre laser, this result being an argument to confirm the relevance of the research. The efficiency of the cutting process was studied according to the material parameters and the characteristics of the laser radiation by Pocorni. The author defined the cutting efficiency according to the area charged with an energy consumption of 1 Joule. The conclusion of the study is that the fibre laser has a better efficiency than the CO_2_ laser on thin sheets [42]. We find a similar conclusion in Shin. For thick carbon steel sheets, the author recommends oblique underwater cutting to the right. The selected cutting speeds were 40, 15 and 7 mm/min for thicknesses of 48, 59 and 69 mm. The author concluded that the cutting efficiency decreases with increasing thickness of the sheets [43]. Orishich et al. compared fibre and CO_2_ lasers to cutting low-carbon steel and stainless steel. The absorbed laser energy, measured in relation to the volume of material removed, was in the range (11–13) J/mm^3^ for both types of lasers [44].

Several papers have been identified in the literature that present comparisons between CO_2_ laser cutting and fibre laser cutting. Aiming to identify the scope of lasers of various types, Fomin et al. compared the quality of the cut. The authors used low-carbon steel and stainless-steel sheets with a thickness of 3–10 mm. They used fibre lasers and CO_2_ lasers with oxygen or nitrogen. The authors concluded that the CO_2_ laser is more efficient for laser-oxygen cutting, and the fibre laser is more suitable for neutral gas cutting [45]. Another comparative analysis was performed by Stelzer et al. on stainless steel AI-SI304. Analysing the results of fibre and CO_2_ laser cutting, the authors identified a sudden increase in surface roughness. The difference in roughness is manifested for material thicknesses between 4 and 6 mm in the case of the fibre laser, and between 8 and 10 mm for the CO_2_ laser [46]. Sołtysiak et al. compared the functional parameters and the quality of the surface generated by laser cutting. During the experiments, 6 mm thick S235JR steel plates with two different types of laser were cut. The results showed that fibre laser cutting gives a more accurate surface at the same linear energy consumption of 55.4 kJ/m. According to the authors, a major impediment is the high purchase price of fibre lasers [47]. Zaitsev et al. simulated the melting dynamics of inert gas laser cutting. The authors studied the removal of molten material and the energy absorbed on the cutting surface for steel sheets with thicknesses of 1, 5 and 8 mm cut with CO_2_ laser and fibres. The authors conclude that the intensity distribution of the absorbed energy is the same for both types of lasers. The intensity of the radiation varies depending on the thickness of the material and the overheating of the walls remains a problem with the fibre laser [48].

The research aims to investigate the working parameters of CO_2_ laser cutting of Hardox 400 steel so that the cutting efficiency is maximum. The experimental research was designed to statistically and analytically evaluate the influence of input sizes on cutting efficiency. Another contribution of the authors is the establishment of a set of relationships to characterize the cutting efficiency. A different area of research was the determination of linear energy in laser processing. The scientific contribution of the paper was based on the calculation of melting speed and melting depth, in order to provide explanations for the physical phenomena that occur in the interaction between laser and material.

## 3. Materials and Methods

The higher number of input parameters tested, conducted to the better the chances of correctly identifying the factors that decisively influence the cutting process with a CO_2_ laser beam. However, the choice of several input factors leads to a slowing down of the decision-making process, due to an increased number of tests to be performed. The choice of the three input parameters (speed, pressure and power) and specific variation intervals was based on preliminary experimental studies and results demonstrated in the literature. The hierarchy of the three parameters was made on the basis of a full factorial design, to ensure a direct connection with the energy used in the studied system. The selection of the parameters, complied with the principle that each parameter must be able to accept all the associations of the tested values, with those retained for the other parameters. In addition, the results obtained are a continuation of other results already published. 

The chemical composition of the material used in the experiments is presented in Table 1. The main mechanical characteristics are yield strength R_e_ = 1000 N/mm^2^, tensile strength R_m_ = 1250 N/mm^2^ and hardness in the range (370–430) HBV [7].

A 6 kW laser was used for the experiments. The auxiliary gas used was CO_2_. A conical profile nozzle was used. The diameter of the nozzle at the outlet was 1.5 mm. Table 2 contains the parameters used in experiments with CO_2_ laser cutting.

**Figure 1:**
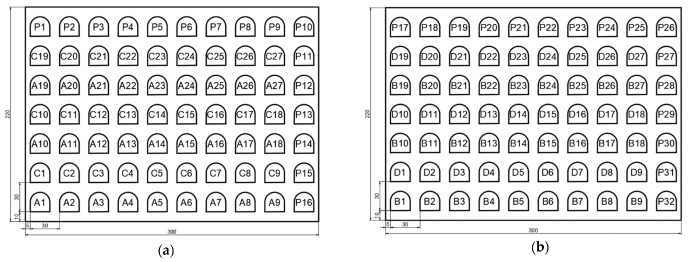
Cutting plan. (**a**) Part 1–70; (**b**) Part 71–140.

The analysed semi-finished product had the dimensions: length 300 mm, width 220 mm, thickness 8 mm, perimeter 1040 mm, side surface 8320 mm^2^ and mass 5.18 kg. The semi-finished product was in standardized form, available from the manufacturer with thicknesses between (2–30) mm. In order to achieve a complete factorial model, the cutting plan was made in Figure 1.

**Figure 2:**
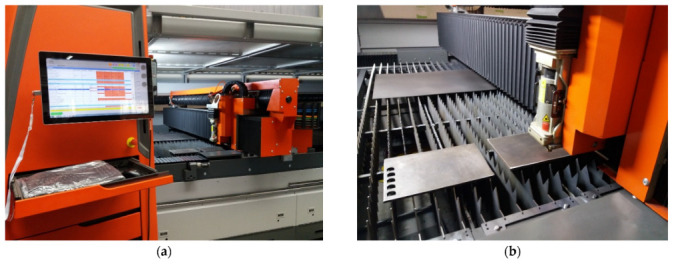
Images with By Autonom 4020 Laser Installation. (**a**) Before processing; (**b**) During processing.

The experiments were performed on a By Autonom 4020 laser cutting machine (Bystronic Laser AG, Niederönz, Switzerland), shown in Figure 2. The factorial design consisted of 27 tests, followed by 4 replicates, for verification and validation. Before starting the experiments, the plate was carefully analysed in accordance with EN 10 163-2. For the calibration of the installation, several tests were performed to determine the range of values in which the processing will take place. The settings of the installation have been made to ensure the continuous conduct of the cutting experiments. The design was built consisting of all possible combinations of parameters.

**Figure 3:**
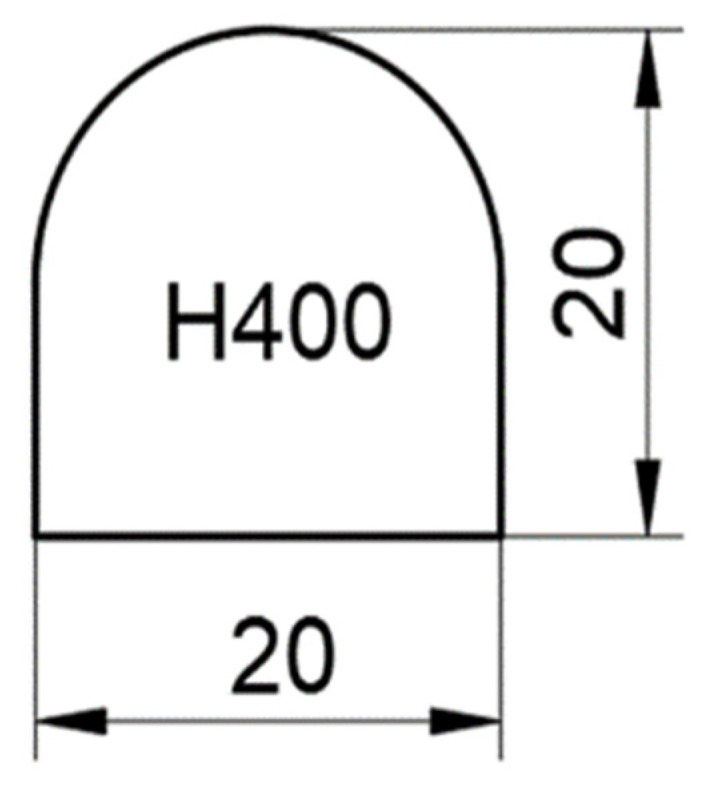
Piece dimensions (mm).

The experimental project consisted of laser processing of Hardox 400 semi-finished products in order to obtain 27 × 5 = 135 pieces. The profile of a part consists of 20 mm long, and 20 mm high, with 3 straight cutting profiles and a curved one (Figure 3). 

The correct choice of cutting parameters is a mandatory condition for obtaining correct results. Initially, a single central test point was established against which all independent tests were performed. After analysing the tests, the reference values of the input quantities were established as follows: cutting speed 1800 mm/min, laser power 5000 W, and assist gas pressure 0.50 bar. The processed pieces were divided into series. The order of parts processing can be seen in Figure 1. The measurement of the parts was conducted electronically. Experimental data were statistically processed with Minitab software, version 19 and Graph software, version 4.

The study presents a comparative analysis of the effect of the parameters, given by statistical prediction with the mathematical model, calculated by the regression method. Independent experiments were performed to determine the functional link between variables. The dependent variables were linear energy and cutting efficiency, and the independent variables were laser power, pressure and speed. The variation of three independent parameters determines the influence on the linear energy and the cutting efficiency.

The Hardox 400 steel cutting efficiency is calculated based on cutting speed, material thickness, and laser power. Two independent parameters vary (speed and power), and one is kept constant (the pressure of the assistant gas) to bring enough information about 1 KJ of laser energy consumed, the volume charged by this energy consumption.

The DoE allowed the design, development and obtaining of experimental data. The laser cutting experiments were designed according to a complete factorial plan containing 3 parameters × 3 values × 3 levels, meaning 27 independent experiments, in which at least one parameter changes. The DoE supports research to save energy, materials and reduce costs. At the same time, it prepares a set of experiments to obtain key information about how influencers influence responses. Four sample tests (4T) were performed to select the core parameters (laser power = 5000 W, auxiliary gas pressure = 0.50 bar and cutting speed = 1800 mm/min). Compared to these values, the minimum and maximum values of the input parameters were chosen by successive tests.

The cutting plan was made electronically with Bysoft 7 and loaded into the laser machine program. The ByVision interface provided the information needed for the manufacturing process (material, cutting and working parameters). The role of the cutting experiments was to identify the threshold energy for which the cut occurs. The material was subjected to the cutting operation under various conditions. The experimental plan describes how the cutting parameters run in order to obtain the lowest energy for which the cutting is performed. The experimental design can be considered appropriate by identifying the variation of the efficiency of the cutting process. Finding the critical energy to process Hardox parts is necessary in the manufacturing process. Cutting efficiency indicators are physical process quantities determined from input data. Determining these indicators can help reduce the amount of molten and discharged material that produces the cut. The thermal erosion of the part allows us to establish the limits of variation of the energy and the efficiency of cutting according to certain factors of influence. Energy is the physical quantity used in industrial laser processing.

## 4. Results and Discussions

### 4.1. Values of Output Responses

Table 3 contains the measured values of the response variables for linear energy and cutting efficiency in relation to the input variables. The linear energy value was measured in the range (154.7–180.0) J/mm, based on the 27 experimental tests. Similarly, the cutting efficiency varied in the range (44.4–51.7) mm^2^/KJ.

The maximum cutting efficiency of 51.6 mm^2^/KJ is obtained at minimum laser power and maximum speed. The maximum melting efficiency of 20.6 mm^3^/KJ is obtained at the maximum speed and minimum power for part 3. The maximum linear energy of 176.4 J/mm, corresponding to the energy consumption of 1 J to cut 1 mm of material, is obtained in parts 10, 13, and 16. The research indicates certain parts with calculated physical sizes, which can be chosen to improve the cutting process in other subsequent experiments, which will increase the cutting efficiency, as well as the dependence of the melting efficiency on the pressure of the gas.

The laser produces concentrated energy for the local melting of the cut material. It is found that the melting efficiency is maximum in processing conditions at a cutting speed of 1900 mm/min while the laser power is set at 4900 W. It is observed that for this processing mode the melting volume is 20.6 mm^3^ corresponding an energy consumption equal to 1 KJ.

The assist gas pressure does not affect the melting efficiency. Selecting the cutting speed at 1700 mm/min at the same time as the laser power of 5100 W leads to the lowest amount of molten material, the cutting process is uneconomical. It can be appreciated from an energy point of view that the consumption is high when the cutting speed is minimum and the power is maximum. In these conditions, a volume equal to 13.3 mm^3^ melts. Due to the long interaction time between laser and material, the amount of energy consumed by 1KJ is transformed into local heat that is dissipated by thermal conduction.

Table 4 shows an average melting efficiency of 16.2 mm^3^/KJ under processing conditions with the pressure kept constant at 0.5 bar, being the lowest for the first 9 parts. Conclusive results regarding the melting of the material are derived from the experimental data. At a pressure of 0.45 bar, a speed of 1900 mm/min and a power of 4900 W, an efficiency of 20.6 mm^3^/KJ is obtained, and at a pressure of 0.5 bar, a speed of 1900 mm/min and a power of 5100 W, the efficiency is 19.8 mm^3^/KJ. It follows that the maximum cutting speed will ensure the lowest energy consumption.

Gas pressure is a state parameter being considered one of the main parameters of the laser processing process. The role of pressure in laser cutting is to remove the melt, to protect the laser beam and the lens. Pressure is not in the relationship between defining energy indicators and laser cutting efficiency. This result led to the constant maintenance of the pressure at the average value. This explains why the effects of pressure are statistically insignificant.

The energy of the cutting process characterization is conducted with the help of dynamic sizes. The calculation was made to estimate the energy consumed when cutting the full factorial experiment planned. There are two energy indicators that shape the laser processing process. Thus, the linear energy of each piece was added together and the total linear energy E_l_ = 4509.2 J resulted. Given that the experiment has n = 27 samples, with the contour length estimated at an average of l = 100 mm, the total energy required to cut the parts of the experimental design is obtained:

(4) $${E=E}_{l}\cdot n\cdot l.$$

Another energy indicator is the breakthrough energy (E_p_), having the significance of point energy, calculated according to the relation:

(5) $$E_{p}{=E}_{l}\cdot \mathrm{Kerf}.$$

The values E_l_ = 4509.2 J and Kerf = 9.2 mm (corresponding to the 27 pieces in series 1) were used in the relationship to estimate the punching energy. As a result of 27 cutting operations, a breakthrough energy of 41,485.2 J was consumed.

The energy of the laser spot (E_s_) is determined by the relationship:

(6) $$E_{s}=\frac{E_{p}}{\mathrm{Number}\;\mathrm{parts}}=1536.4\;J.$$

The melting area (A_m_) is deduced from the energy density (ρ_e_ = 128.04 × 10^5^ J/cm^2^), the breakthrough time (τ = 0.7 s) and the laser power (P = 5000 W):

(7) $$A_{m}=\frac{\;P\cdot \tau }{\rho_{e}}.$$

Table 5 shows the characteristic quantities deduced from the energy calculation required for laser processing.

During processing, a thermal effect occurs on the semi-finished products, due to the energy of the laser radiation, which is transformed into local heat. During cutting the laser emitted a pulse of duration (2.2–2.4) s. The maximum energy emitted for cutting a part is 4.5 × 10^5^ J. Certain physical quantities that can be estimated from the interaction of the laser with the steel can be estimated from the heat equation. The material heats up due to the thermal energy received from the laser spot. The elementary expression of heat is given by the relation [49]:

(8) $$\mathrm{dQ}=m\cdot c\cdot \mathrm{dT}=\rho \cdot V\cdot c\cdot \mathrm{dT}=\rho \cdot V\cdot c\cdot (\frac{\partial T}{\partial x}\mathrm{dx}+\frac{\partial T}{\partial t}\mathrm{dt}).$$

The heat flux dQ/dt is given by Fourier’s law, vc = dx/dt represents the rate of heat penetration into the material, and when the temperature reaches melting dTm = 0. The surface area is equal to the area of the spot, and by approximation, the volume of the melt has a diameter equal to the melting depth (z_m_). With these conditions and performing the calculations, the relation for the melting depth is obtained:

(9) zm=(38·d2·zc)13,

where z_c_ is the depth of heat penetration into the material depending on the thermal diffusion and the duration of the laser pulse z_c_ = 2·(β·τ)^1/2^ [50]. The data required to determine the depth of penetration were grouped in Table 6.

### 4.2. Analysis of Linear Energy and Cutting Efficiency

**Figure 4:**
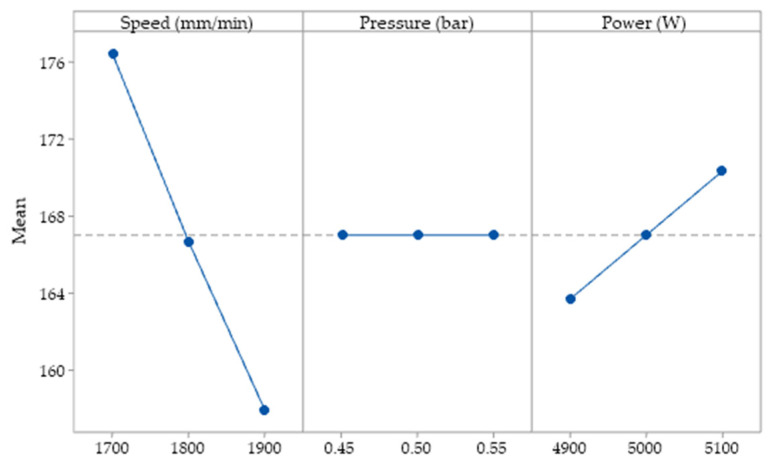
Main effects for linear energy.

In order to establish the influence of the parameters considered in the research on linear energy, the experimental data were subjected to statistical analysis. The impact of input variables on linear energy is shown in Figure 4.

The main effect graphs indicate the response of the cutting parameters at each level considered. The Y-axis of the graph represents the linear energy, and the X-axis represents the input parameter. From Figure 4 it can be seen that an increase in the scanning speed has the effect of a decrease in the values of the linear energy. When the laser power is selected to values above 4900 W, an increase in power per unit of contour is obtained from a line graph. The raising of the power of the laser results in a greater transfer of laser energy to the material, thus accumulating local heat, which leads to a more molten material. The increase in cutting speed has led to lower values of linear energy, following a reduction in the interaction time between the laser and metal. Constant maintenance of the linear energy was observed at the variation of the gas pressure values. Increasing or decreasing the gas pressure has no effect on linear energy. At the minimum speed, we obtain maximum linear energy. At average values of the input parameters, the response remains constant. At maximum cutting speed, a shorter machining time and less energy are obtained. 

**Figure 5:**
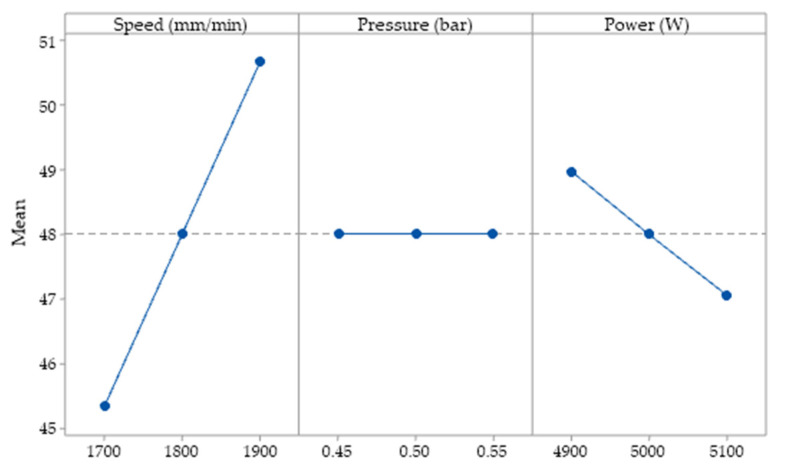
Main effects for cutting efficiency.

Figure 5 shows the impact of input factors on cutting efficiency. From the results obtained it can be seen that, when the speed has higher values, there is an increase in cutting efficiency after a straight line. The phenomenon is more pronounced after exceeding the speed of 1800 mm/min. The assist gas pressure has a discontinuous influence. A pressure between (0.45−0.5) bars reduces the melting efficiency. Above 0.5 bar the cutting efficiency increases. It can be seen that when the laser power is between 4900 and 5000 W the cutting efficiency decreases. Further increasing the laser power above 5000 W the cutting efficiency is better.

**Figure 6:**
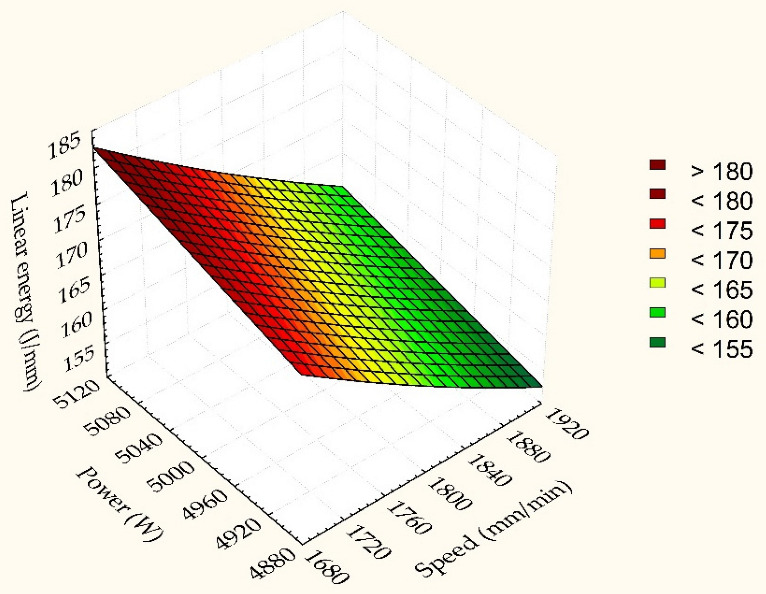
Linear dependence of linear energy as a function of speed and laser power.

Cutting efficiency is maximum when the speed is set to the maximum value. The analysed response increases linearly with speed. The laser power linearly decreases the cutting efficiency when is set between the minimum and maximum range. Figure 6 indicates that the more pronounced effect on cutting is due to laser scanning speed. The second effect is imprinted by the power, followed by the assist gas pressure. The maximum speed ensures the best result of the cutting efficiency. The combination of minimum power and maximum speed produces maximum efficiency. The pressure factor has a low influence on the output parameter. The effects due to the independent parameters are linear. By adjusting the working parameters, we ensure better control of the process.

### 4.3. Linear Predictive Model for Linear Energy and Cutting Efficiency

**Figure 7:**
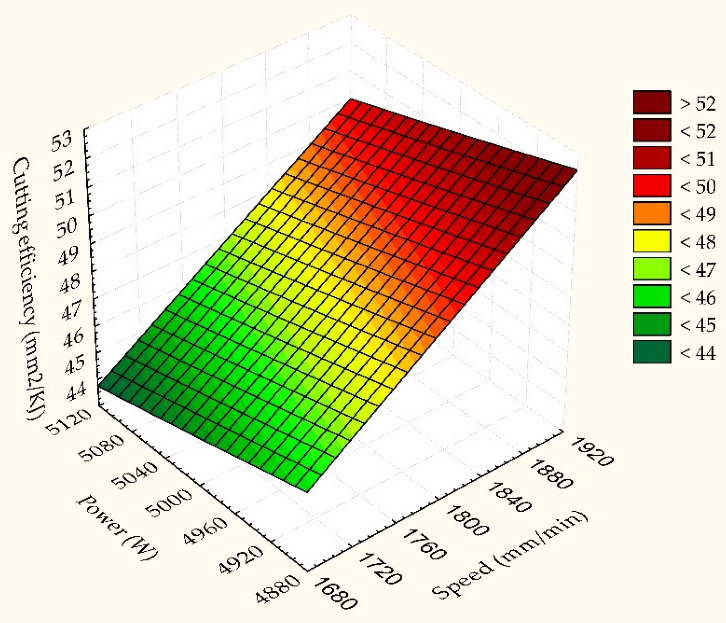
Linear dependence of cutting efficiency as a function of speed and laser power.

The model is shown in Figure 6 and Figure 7 shows that, during CO_2_ laser cutting, the laser power has values in the range (4900–500) W. If a value of the laser power is chosen outside this range, is observed a deterioration of the linear energy. The simultaneous variation of two parameters of influence (cutting speed, power) on the linear energy and the cutting efficiency, if the pressure gas is constant, contributes to the determination of the optimal combination formed by the predictive factors. The linear predictive graph was obtained with the method of response surfaces (RSM). The response area method used in statistics (RSM) shows the relationships between input variables and responses. The central idea of the method is to obtain an optimal answer based on a set of designed experiments [51]. It looks like a 2D surface that contains combinations between cutting speed and laser power. The interaction of the two influencing factors leads to the following results:

- If the cutting speed is in the range (1860, 1900) mm/min and the laser power has values in the range (4900–5000) W results in minimum linear energy.
- Gradually changing the cutting parameters, relative to these values, increases the linear energy and decreases the cutting efficiency. An increase in energy means a high consumption and production cost. The maximum values of the linear energy are obtained in conditions of minimum speed and maximum laser power.

The maximum values of the cutting efficiency are obtained at maximum speed with minimum laser power. From Figure 6, it is observed that the linear energy decreases more strongly with the speed of cutting.

In the case of cutting efficiency (Figure 7), the decrease is also more synergistic with the processing speed. The two responses seen as quality indicators vary linearly across a flat surface. It consists of different colours that indicate the strength of the output responses.

The cutting efficiency is kept to a minimum when the laser power is selected at maximum while the cutting speed is set to a minimum. Maximum cutting efficiency is obtained under conditions of minimum power at maximum speed. The slope of the graph is more inclined to be influenced by the cutting speed. As a result, speed is the most influential parameter.

Using the regression analysis, the mathematical relationships for each response variable were obtained. The regression equations are presented in relations (11) and (12), respectively:

(10) $$E_{l}=167.18\;–\;0.09\cdot V+0.03\cdot P.$$

It turns out that the most important factor that determines linear energy is the cutting speed because it has a correlation coefficient of 0.09, higher than that of the power of 0.03. The effect induced by the increase in cutting speed is to decrease the linear energy. The coefficient of the model is 167.18. The power of the laser is a factor that causes the linear energy to increase. Cutting speed acts to reduce linear energy by providing a lower amount of laser energy.

(11) Ec=48.019+0.026·V – 0.009·P.

In the case of cutting efficiency, the power acts in the sense of decreasing it, and the cutting speed determines the increase of the output variable, due to the sign-in relation (12). The cutting speed becomes dominant in this case because it has a correlation coefficient of 0.026, higher than the laser power of 0.009. The coefficient of the model is 48.019. The two regression equations calculate exactly the two analysed answers.

### 4.4. Quadratic Predictive Model of Linear Energy and Cutting Efficiency

**Figure 8:**
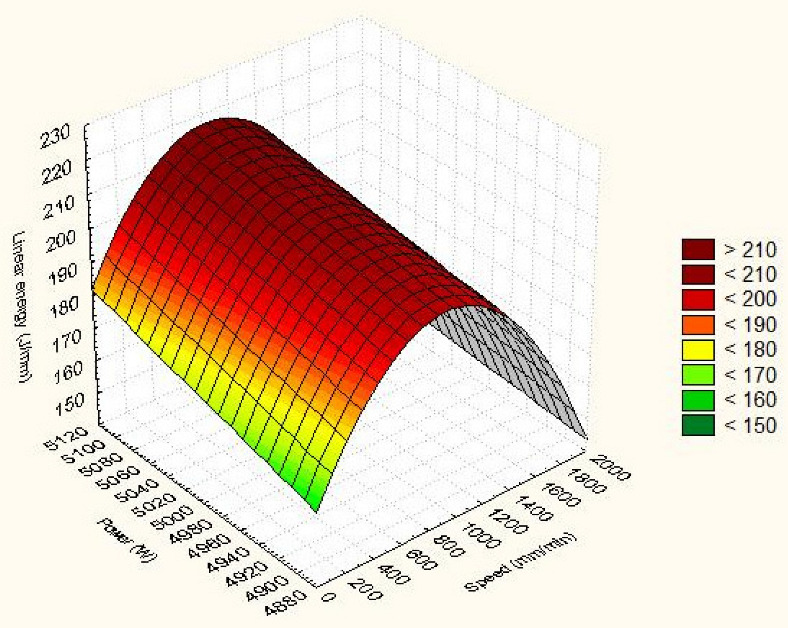
Quadratic dependence of linear energy on power and speed.

**Figure 9:**
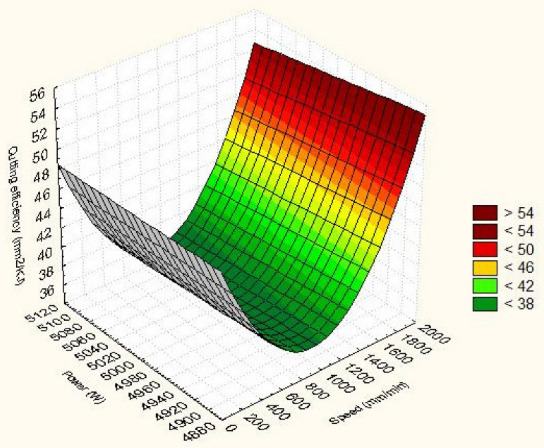
Quadratic dependence of cutting efficiency and speed.

The 3D surface in Figure 8 and Figure 9 provides a conclusive frame for anticipating how the input parameters considered affect the linear energy and the cutting efficiency of Hardox 400 steel parts.

Figure 8 shows that the linear energy is minimal when we set the cutting speed to the maximum value while the laser power is minimal. As the speed decreases and the laser power increases relative to the minimum point, there is a square increase in linear energy. The shape of the surface of the linear energy is a paraboloid. It is observed that speed deforms the quadratic surface faster than power. In the conditions of setting the maximum power and the speed at medium, it results that the linear energy is the highest.

The estimated quadratic model of linear energy, using the regression method is shown in Figure 8:

E_l_ = −302.162 + 0.215·V + 0.109·P − 4.997 × 10^−5^·V·V − 2.576 × 10^−5^·V·P − 2.967 × 10^−6^·P·P. (12)

The maximum value of linear energy 176.4 J/mm is obtained for cutting speed 1700 mm/min and laser power 5000 W. The correlation coefficients of square laser speed and power are very small.

Under these conditions, the correlation coefficient of the square speed is higher than that of the laser power. The relation shows that the coefficient of linear velocity 0.215 is higher than that of laser power 0.109. It turns out that cutting speed is the most influential factor. The linear interaction between laser speed and power decreases the linear energy. The coefficient of the model is −302.162.

Maximum cutting efficiency is obtained when the laser power is minimal while the cutting speed is maximum. Gradually changing the cutting parameters from this critical point decreases the cutting efficiency. It is also found that speed is the principal factor that changes the cutting efficiency. The decrease is stronger when the speed is acting because the efficiency is reduced faster. The increase in energy has the effect of gradually decreasing the cutting efficiency. If the speed decreases progressively, a larger amount of molten and removed material is ensured.

The estimated square model of the cutting efficiency, using the regression technique, has the shape from Figure 9:

E_c_ = 117.673 − 0.006·V − 0.025·P + 1.500 × 10^−5^·V·V − 4.262 × 10^−6^·V·P + 2.366 × 10^−6^·P·P. (13)

The coefficient of the model is 117.673. The correlation coefficients of the quadratic input variables are very small. However, the value of an input parameter is in the thousands, so the term power, speed or power–speed interaction becomes significant in calculating efficiency. A coefficient of cutting speed higher than that of laser power is observed from the quadratic relation. It turns out that the speed is the principal parameter. In addition, the cutting speed and power of the quad laser increase the cutting efficiency.

The comparison of linear and quadratic predictive models shows approximate results of linear energy under conditions of maximum power and low speed. The quadratic model is superior to the linear model. Linear energy decreases at minimum power and speed. Appropriate results of cutting speed at maximum value with minimum laser power were found. The two models are suitable for the study performed.

## 5. Results

### 5.1. Lagrange Interpolation Method

To validate the results, we aim to find the Lagrange interpolation polynomial that establishes a link between the cutting efficiency (output variable) and a controllable parameter of the cutting process (speed). The points at which this study is performed and the mathematically calculated output responses with the experimental data can be established (Table 7).

The Lagrange (L) function is constructed from the values of the input-output parameters. It comes to the mathematical relationship:

(14) $$L(x)=\frac{46.2\cdot (x\;-\;1800)\cdot (x\;-\;1900)\;-\;97.4\cdot (x\;-\;1700)\cdot (x\;-\;1900)+51.7\cdot (x\;-\;1700)\cdot (x\;-\;1800)}{20,000}.$$

The result is a mathematical relationship that approximates the connection between cutting efficiency and speed:

(15) $$E_{c}=0.0000265\cdot V^{2}\;-\;0.06815\cdot V+85.52.$$

In the functional efficiency–speed relation, the correlation coefficient of the square speed is 0.0000265, and the linear speed coefficient is 0.06815. These coefficients have low values, but the square term increases due to the high speed. The coefficient of the Lagrange interpolation model is 85.52. Checking the relationship is important as it ensures the stability of the cutting process. Substituting each value of the level of the cutting speed in the quadratic relation obtains the values from Table 8.

Table 8 shows the values of the cutting efficiency obtained with the mathematical method and the Lagrange interpolation method. There are very small calculation errors between the data obtained by the two mathematical models. The best efficiency is obtained with the maximum cutting speed. Lagrange interpolation renders a precise polynomial between efficiency and speed that can be used to make the process economical. Physically, increasing the cutting speed means better absorption of laser energy by the material. Under these conditions, a suitable and efficient technological cutting process is obtained. The very small error indicates a good match between the studied models.

The verification with the statistical regression model is conducted by replacing in the quadratic polynomial (V,P) (relation (14)) the laser power P = 4900 W. In these conditions, the cutting efficiency with the statistical method varies according to the cutting speed by the relation:

(16) $$E_{c}=0.000015\cdot V^{2}\;-\;0.0269\cdot V+49.$$

The relations are given by Lagrange and statistically have correlation coefficients of the same order of magnitude, and the signs of the correlation coefficients are identical. In these conditions, it can be appreciated that the two models are appropriate and fit. Analytical relationships determine the efficiency of cutting and can be used in industrial activity.

Table 9 shows the cutting efficiency obtained with the Lagrange interpolation method and the regression method from the statistical software used. Approximate values of cutting efficiency are obtained. The relative errors are very small; it turns out that the two models are suitable for the study performed. The error polynomial results from relations (16) and (17):

(17) $$E_{c}^{\mathrm{Error}}=0.0000115\cdot V^{2}\;-\;0.04125\cdot V+36.52.$$

By replacing the cutting speed in the error polynomial, an efficiency modulus of 0.37 is obtained. The deviation of the cutting efficiency is 7.9%. The data calculated by different methods are approximate. It turns out that the two models (Lagrange, statistically) are convergent. The most accurate efficiency response is given by the statistical model, the value obtained being 52.04 mm^2^/KJ. Basically, we can cut an area of 52.04 mm^2^ while consuming 1 KJ of laser energy. Another possibility to optimize the cutting parameters is obtained from the criterion analysis. It contains a ranking of the pieces on each indicator. Table 10 classifies the processed parts according to the numerical values for linear energy, cutting efficiency and melting efficiency. The first three tracks from the total of 27 in the first series were retained. It was found that track 3 has the lowest power consumption and the best efficiency.

One of the most effective ways to establish the results of research is to appreciate it from a monetary point of view. Establishing energy costs helps to substantiate decisions related to the study process. Only the energy consumption involved in laser cutting was considered, due to the fact that the research is oriented in this direction. Other material, wages and other consumption are not calculated. The input data required to determine energy consumption were centralized in Table 11. In the first column of the table, the elements of energy cost were identified, in order for this to be expressed in monetary units. The second column contains the calculation relationship for each energy consumption. The last column contains the numeric values obtained. Consumption was expressed in euro/hour, to make it easier for users to understand. The exchange rate used for the conversion from lei (Romanian currency) to euro was an average value recorded between May 2021 and May 2022 [52]. The average tariff for electricity consumed was determined for a non-household consumer, average voltage level, taking into account the offers of the main operators in the Romanian energy market and other elements such as green certificates, the contribution for high-efficiency cogeneration and excise. The calculation resulted in an average tariff of 0.29 euro/kWh. The power factor (efficiency) considered in relationships is 80%.

### 5.2. Validation of Results

The contribution of the input parameters was evaluated using the ANOVA method for each response variable. R-sq. coefficients and R-sq. (Adj.) were used to show the importance of the data and the percentage in which the input parameters influence the response. Table 12 shows the results of the ANOVA linear energy technique. The ANOVA analysis was performed at a 95% confidence level. Corresponding to this confidence level, the value of p should be less than 0.05, to indicate that it has a significant effect on the selected answer [53,54]. Table 12 shows that speed and laser power have a significant impact on linear energy because the p-values are less than 0.05. The assist gas pressure has no significant influence, the *p* value being 1.00 > 0.05.

The values obtained are R-sq. = 99.97% and for R-sq. adjusted, 99.96%. The very small difference between the two values shows that the model obtained is suitable for linear energy. DF represents the degrees of freedom, SS is the sum of the squares of the regression and the errors, MS is the mean of the sum of the squares and F is the average Fischer [53]. As the p is found to be very small and the Fischer mean is high, there is a strong relationship between response and predictors. From Table 12, it can be seen that the speed input was the highest at 88.53%, followed by the power at 11.43%. The gas pressure is not significant for the model obtained. The errors are negligible, their weight being 0.002%, which indicates that the results can be used for future predictions.

**Figure 10:**
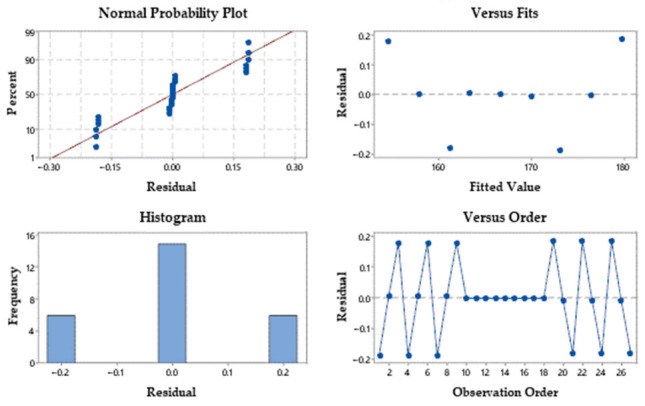
Residual plots for linear energy.

Figure 10 graphically shows the residual plots for linear energy (normal probability plot, versus fits, histogram and versus order). Taken together, the residual diagram tests show a good concordance similar to the linear energy, which satisfies the results of ANOVA.

In order to calculate the significance of the input variables in the proposed model, the ANOVA method was used for cutting efficiency. The results are presented in Table 13. The ANOVA results for cutting efficiency show that the speed and power have a significant influence because the p values for the two parameters were 0.0001, values lower than 0.05. Values for R-sq. and R-sq. (Adj.) shows that the proposed model matches the experimental data. The difference between the values is very small (less than 20%). Table 13 shows that the cutting speed (88.50%) has the greatest influence on efficiency, followed by laser power (11.47%). The pressure of the gas has no influence on the cutting efficiency, the value of p is 1.00. The error rate is negligible at 0.02%, indicating that the proposed model can be used for future predictions.

**Figure 11:**
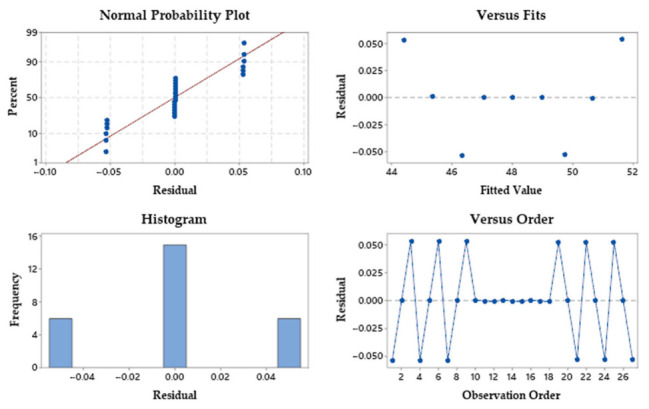
Residual plots for cutting efficiency.

Figure 11 presents the residual plots for cutting efficiency. All tests of the residual diagram show a good concordance, similar to the cutting efficiency, which satisfies the ANOVA results. This also presents a good match of the proposed model for cutting efficiency.

Theoretical research focuses on the movement of the melt in the direction of two cutting fronts on the cut surface. At the initial moment, the drop has mass m_0_ and the initial velocity ω_0_ = 0. After a very short time, the drop has mass m and velocity ω. In an infinitesimal time dt the melt loses the infinitesimal mass dm, which represents the mass of the metal droplets with velocity u.

In this case, there are two varying physical sizes, mass and speed. By applying the law of conservation of momentum, where at a given moment the initial impulse (p_i_) is equal to the final impulse (p_f_), one can estimate the relationship between the velocity of the melt and the velocity of the hot droplets. This result can cause the kinetic energy of the melt drop system to release heat into the slit.

(18) $$p_{i}=m\cdot \omega ,$$

(19) $$p_{f}=(m\;-\;\mathrm{dm})\cdot (\omega +d\omega )+u\cdot \mathrm{dm},$$

(20) m·dω=(ω - u)·dm,

(21) $$\frac{\mathrm{dm}}{m}=\frac{d\omega }{\omega \;-\;u},$$

(22) $$\int_{m_{0}}^{m}\frac{\mathrm{dm}}{m}=\int_{0}^{\omega }\frac{d\omega }{\omega \;-\;u},$$

(23) $$n\frac{m}{m_{0}}=\ln\frac{u\;-\;\omega }{u},$$

(24) $$\omega =u\cdot (1\;-\;\frac{m}{m_{0}}).$$

The droplets move in the opposite direction of the melting (m < m_0_). The speed of melting is slower than the speed of the droplets (ω < u). The variable mass of the melt affects the roughness. If the mass decreases, the roughness at the exit of the slot is lower. The width of the cut is another variable parameter due to the decrease in the volume of the melt. There is a dm/dt mass flow that prints a stream of sparks leaving the slot. The energy resulting from the transformation of kinetic energy into heat strongly heats the cut surfaces. With the absorbance of the laser energy by the material, the melt is formed, subsequently removed and damaged by the flux formed by the assistant gas. The velocities of the expelled droplets and the melt enter the expression of the kinetic energies that determine the energy equation.

The total energy (Q) required to heat the target under the action of the Gaussian laser pulses emitted by the CO_2_ laser having a Gaussian intensity distribution in the laser spot, is presented in the following relation [55,56]:

(25) $$Q=2\cdot \pi \overset{a}{\underset{0}{\int }}\overset{\tau }{\underset{0}{\int }}I(r,t)\cdot r\cdot \mathrm{dr}\cdot \mathrm{dt}.$$

Following the calculations, the authors propose the following relation for heat estimation:

(26) $$Q=2\cdot \pi \overset{a}{\underset{0}{\int }}\overset{\tau }{\underset{0}{\int }}I_{0}{\cdot e}^{-\;(\frac{r^{2}}{a^{2}}+\frac{t^{2}}{\tau^{2}})}\cdot r\cdot \mathrm{dr}\cdot \mathrm{dt},$$

where I_0_ maximum intensity, r radial coordinate, a radius of the circular surface, τ duration for the laser intensity to reach the value I_0_/e, t duration for the spot radius to reach the value r. Solving the double integral is conducted with the help of Euler’s formulas.

Heat is equal to the amount of energy transferred from the laser spot to the metal by absorbency. Laser energy transfer is conducted with laser radiation. Following the interaction of radiation produces by laser with the steel, a local convective heat transfer occurs followed by another heat transfer through thermal conductivity. The amount of heat is distributed through the metal following the conduction of electrons, the collisions between electrons and ions in the network and the vibration of ions. Melting and heat affect parts and the working environment. The heat resulting from the laser spot is used for the operation of separating the piece from the waste plate. The way the thermal energy is used explains the processing conditions of the parts. Based on these considerations, other directions of research can be identified. One of the guidelines is to determine dimensional accuracy and expansion. Another direction is to apply the calculation relationships, established by the authors, to other materials or to other laser processing processes.

Cutting efficiency is an energy indicator that shows how many mm^2^ can be cut with 1 KJ laser energy. Linear energy is another indicator implemented to estimate the energy required to debit the unit of contour. Physically, linear energy can be estimated to be equal to the ratio of laser power to cutting speed. Therefore, it is possible to calculate the energy that erodes and cuts the material. The light resulting from the laser–metal impact is due to the collision between the laser photons and the metal electrons resulting in reflected photons and photoelectrons that generate radiation with different frequencies. The laser energy method is based on the concentration of radiation and the appearance of a melt that reflects and absorbs light.

Laser energy savings mean a decrease in electricity consumption. Photons and electrons resulting from the interaction of laser–metal radiation affect organisms. The laser energy produces heat and radiation. As a result, there is a need to identify the most suitable input parameters that produce limited energy so that the cut can be made. The studied indicators ensure a technological process that minimizes energy, increases the safety of processing and generates quality indicators. Statistical research identifies that processing efficiency and linear energy are strongly influenced by speed and power. Instead, the mathematical model indicates the main factor as the cutting speed. From an energy point of view, it is clear that laser speed and power are decisive factors for optimization and efficiency.

The mathematical relations (11)–(14) for linear energy and melting efficiency were obtained statistically. The function that describes the variation of the answer (linear energy and melting efficiency) can be represented graphically according to two predictors (speed and power). The shape variation of the function can be linear (L) or quadratic (Q), the graph being a flat surface or a quadratic surface. Mathematical relationships are determined from experimental data (Table 3) by regression analysis. To validate the mathematical relationships, the value of the minimum laser power and the maximum speed obtained in the cutting experiments on Hardox steel were replaced and the linear energy and cutting efficiency were determined according to Table 14.

Hardox400 has a chemical structure that can absorb CO_2_ laser energy well. If the laser energy increases, it means that the laser power factor has increased, which produces a more intense local heat for melting, decreasing the efficiency of the process. It turns out that increasing the energy of the laser will increase the electricity consumption and manufacturing costs of the parts. When the radiation interacts with the metal, the thermal energy that the laser possesses is transformed into heat. The quality characteristics of the part will depend on the energy indicators. In addition, if we evaluate the cutting speed, it is found that this influencing factor will significantly reduce the heat transfer to the material when the speed increases.

These predictors (cutting speed and laser power) must be selected and controlled in order for the cutting efficiency to improve. Following the experimental analysis (Table 3), it was found that the best cutting efficiency is obtained when the laser power is 4900 W at the same time as the cutting speed at 1900 mm/min. This study shows a very good match between the experimental model and the statistical mathematical model. For these reasons the authors propose a symbolized indicator f, determined as the ratio between f = V/P to express the efficiency of laser cutting (Table 15).

Combining the best minimum power with maximum cutting speed increases the efficiency of the laser cutting process. Other combinations of power and speed reduce cutting efficiency by increasing processing time and cost.

The laser cutting process can be developed by reducing the energy required for manufacturing and increasing efficiency. The following results emerge from this study:

- five mathematical relationships designed for the mathematical modelling of the laser radiation interaction with the material;
- a mathematical method of estimating linear energy and linear efficiency according to two input parameters (power, speed) when the pressure is kept at a constant value;
- linear and quadratic polynomials for linear energy and cutting efficiency developed by the RSM method indicate, by analysing the correlation coefficients of the influencing factors, that the cutting speed is the significant parameter because it has a higher coefficient than the laser power;
- the graphs RSM (L) and (Q) show that the best cutting efficiency is obtained in conditions of minimum laser power and maximum cutting speed;
- the RSM (L) graph shows that in conditions of processing at maximum speed with minimum power, the lowest energy consumption per unit length is obtained;
- the RSM (Q) graph shows that under low speed processing, the power consumption per unit length can be reduced to the lowest length;
- the interaction between laser power and cutting speed decreases linear energy more sharply than cutting efficiency due to higher energy correlation coefficient.

The RSM graphs of energy and efficiency are inverted (plan and paraboloid), easily identifying the answer according to the values of the predictors so that the cutting consumes a minimum of energy.

## 6. Conclusions

The research consists of the analysis of some process parameters of the CO_2_ laser cutting process of Hardox 400 steel plates compared with the response variables. Laser power, cutting speed, and gas pressure were selected as process input variables, while linear energy and cutting efficiency were considered as output variables. The main conclusions drawn from the results are:

1. Maximum cutting efficiency is obtained at minimum laser power and maximum cutting speed of 51.7 mm^2^/KJ.
2. Maximum melting efficiency is obtained at maximum speed and minimum laser beam power for part 3 of 20.6 mm^3^/KJ.
3. The minimum linear energy, which corresponds to the energy consumption of 1 J to cut 1 mm of material, is obtained in parts 3, 6, and 9 of 154.7 J/mm.
4. The linear energy consumed when cutting a part is E_l_ = 4509.2 J.
5. The total penetration energy consumed is 41,485.2 J.
6. The mathematical model determines the cutting speed as an influencing factor, and the statistical model of linear energy shows that the influence of 88.53% has the cutting speed, followed by the power with a percentage of 11.43%.
7. The results of the ANOVA method for cutting efficiency show that cutting speed has the greatest effect on efficiency (88.50%), followed by laser power (11.47%).
8. The statistical calculation presents that the cutting speed determines the extreme values of the linear energy. It also sets a maximum cutting efficiency influenced by speed and a minimum cutting efficiency due to the power of the laser.
9. The predicted linear model shows that at a speed of 1900 mm/min and a laser power of 4900 W the best linear energy and cutting efficiency are obtained.
10. The cutting efficiency was verified by the Lagrange interpolation model and statistics. Similar relationships have resulted, which means that the two models are suitable.
11. The total energy consumption is 9.09 euro/h.
12. Maximum efficiency is obtained at cutting speed 1900 mm/min, gas pressure 0.50 bar, and laser power 4900 W.
13. The 2D analysis of the melting efficiency found that the melted volume with 1 KJ of laser energy consumed increases when the gas pressure has a minimum value (0.45 bar).
14. The 2D analysis found that the energy to cut a linear contour decreases when the speed is maximum 1900 mm/min. Similarly, it was found that the cut surface increases to 1 KJ of laser energy consumed when the speed is maximum.
15. In the 3D RSM (L) the energy on the cut contour is minimal when the maximum speed and minimum power are selected. It is observed that the cutting efficiency becomes maximum for the combination of the maximum speed and minimum power.
16. In 3D RSM (Q) laser cutting the energy per unit length is minimized under processing conditions minimum speed with minimum power or maximum speed with minimum power. Cutting efficiency is maximized under processing conditions with maximum speed and minimum power.

The paper investigated complexly how laser energy influences the cutting process, based on the statistical evaluation of 27 experiments, followed by four replicates, in identifying the most appropriate set of parameters to increase the cutting efficiency. Following this statistically relevant 8 mm thick HARDOX400 study on linear energy and cutting efficiency, cutting speed is the main factor, followed by laser power and assist gas pressure.

## Figures and Tables

**Table 1:** Chemical composition Hardox 400 [7].

Alloying Element	C	Si	Mn	P	Cr	Ni	B	Mo
%	0.20	0.69	1.60	0.024	0.79	1.00	0.004	0.79

**Table 2:** Working parameters.

Parameter (Unit of Measure)	Level 1	Level 2	Level 3
Laser power (W)	4900	5000	5100
Gas pressure (bar)	0.45	0.5	0.55
Cutting speed (mm/min)	1700	1800	1900
Cutting mode	CW		
Piercing time (s)	0.7		
Distance of the nozzle in the piercing phase (mm)	6		
Laser power in the piercing phase (W)	5000		
Focusing position (mm)	1		
Nozzle positioning height (mm)	40		
Nozzle distance in cutting (mm)	1		
Gas pressure in the piercing phase (bar)	0.7		

**Table 3:** Design of experiment. Measured values of output responses.

No. Exp.	Speed (mm/min)	Pressure (bar)	Laser Power (W)	Linear Energy (J/mm)	Cutting Efficiency (mm^2^/KJ)	Melting Efficiency (mm^3^/KJ)
1.	1700	0.45	4900	172.9	46.2	18.5
2.	1800	0.45	4900	163.3	48.9	14.6
3.	1900	0.45	4900	154.7	51.7	20.6
4.	1700	0.5	4900	172.9	46.2	16.1
5.	1800	0.5	4900	163.3	48.9	17.1
6.	1900	0.5	4900	154.7	51.7	15.5
7.	1700	0.55	4900	172.9	46.2	16.1
8.	1800	0.55	4900	163.3	48.9	19.5
9.	1900	0.55	4900	154.7	51.7	15.5
10.	1700	0.45	5000	176.4	45.3	15.8
11.	1800	0.45	5000	166.6	48	14.4
12.	1900	0.45	5000	157.8	50.6	15.2
13.	1700	0.5	5000	176.4	45.3	13.6
14.	1800	0.5	5000	166.6	48	14.4
15.	1900	0.5	5000	157.8	50.6	15.2
16.	1700	0.55	5000	176.4	45.3	13.6
17.	1800	0.55	5000	166.6	48	16.8
18.	1900	0.55	5000	157.8	50.6	17.7
19.	1700	0.45	5100	180	44.4	17.7
20.	1800	0.45	5100	170	47.1	16.4
21.	1900	0.45	5100	161.1	49.6	17.3
22.	1700	0.5	5100	180	44.4	15.5
23.	1800	0.5	5100	170	47.1	16.4
24.	1900	0.5	5100	161.1	49.6	19.8
25.	1700	0.55	5100	180	44.4	13.3
26.	1800	0.55	5100	170	47.1	16.4
27.	1900	0.55	5100	161.1	49.6	17.3

**Table 4:** Average melting efficiency values.

Part Number	Pressure (Bar)	Melting Efficiency (mm^3^/KJ)
1, 2, 3	0.45	17.9
4, 5, 6	0.5	16.2
7, 8, 9	0.55	17.0

**Table 5:** Characteristic quantities.

Theoretical Quantities (Unit)	Total Energy (MJ)	Initial Energy (KJ)	Melting Area (mm^2^)	Spot Energy (J)
Numeric value	12.175	4.148	0.027	1536.4

**Table 6:** Characteristic sizes.

Physical Sizes	Thermal Conductivity (cm^2^/s)	Pulse Duration (ms)	Melting Depth (µm)
Numeric value	10^−5^	2.5	1.5

**Table 7:** Experimental data used in the Lagrange interpolation method.

No.	Lagrange Interpolation	
	Input Parameter	Output Parameter
	Speed [mm/min]	Cutting Efficiency [mm^2^/KJ]
1.	V1 = 1700	f(v1) = 46.2
2.	V2 = 1800	f(v2) = 48.9
3.	V3 = 1900	f(v3) = 51.7

**Table 8:** Cutting efficiency obtained by different methods.

Lagrange Interpolation/Calculated	Speed [mm/min]	Cutting Efficiency (Lagrange) (mm^2^/KJ)	Cutting Efficiency (Relation, Definition) (mm^2^/KJ)	Relative Error (%)
Minimum level	1700	46.2	46.2	0
Medium level	1800	48.7	48.9	0.0053
Maximum level	1900	52	51.7	0.0057

**Table 9:** Lagrange cutting efficiency and statistical calculation.

Speed [mm/min]	Cutting Efficiency (Lagrange) (mm^2^/KJ)	Cutting Efficiency (Statistical) (mm^2^/KJ)	Relative Error (%)
1700	46.2	46.6	0.0079
1800	48.7	49.1	0.0095
1900	52	52.0	0.0007

**Table 10:** Parts classification.

Linear Energy	Cutting Efficiency	Melting Efficiency
3	3	3
12	12	24
27	27	8

**Table 11:** Energy cost calculation.

Energy Cost Element	Calculation Relationship	Value (euro/h)
Laser	21 kVA·0.29·0.8	4.87
Cooling system	11.50 kW·0.29·0.8	2.66
Moving the cutting plant	5.5 kW·0.29·0.8	1.27
Ventilation system	1 kW·0.29	0.29
Total	9.09	

**Table 12:** ANOVA results for linear energy.

Source	DF	SS	MS	F	p	Remark
V	2	1554.38	777.189	37,500.43	0.0001	Significant
p	2	0.00	0.000	0.00	1.000	Unsignificant
P	1	200.83	100.413	4845.08	0.0006	Significant
Error	20	0.041	0.021			
Total	26	1755.62				
R-Sq. = 99.97%, R-Sq. (Adj.) = 99.96%, S = 0.143961						

**Table 13:** ANOVA results for cutting efficiency.

Source	DF	SS	MS	F	p	Remark
V	2	128.068	64.0342	37,485.26	0.0001	Significant
P	2	0.000	0.0000	0.00	1.000	Unsignificant
P	2	16.604	8.3021	4860.03	0.0008	Significant
Error	20	0.034	0.0017			
Total	26	144.707				
R-Sq. = 99.98%, R-Sq. (Adj.) = 99.97%, S = 0.0413310						

**Table 14:** Results obtained.

Type of Verification	Power (W)	Speed (mm/min)	Linear Energy (L) (J/mm)	Linear Energy (Q) (J/mm)	Cutting Efficiency (L) (mm^2^/J)	Cutting Efficiency (Q) (mm^2^/J)
Statistic	4900	1900	154.3	153.9	51.7	51.9
Experimental	4900	1900	154.7	154.7	51.7	51.7

**Table 15:** Efficiency factor calculation.

Predictors	Power (W)	Speed (mm/min)	Efficiency Factor
Case 1	4900	1900	0.38
Case 2	5100	1700	0.33

## Data Availability

The data presented in this study are available on reasonable request from the corresponding author.
